# Effect of Self‐Care Activities on Blood Pressure Control Among Patients With Hypertension

**DOI:** 10.1155/bmri/6623871

**Published:** 2025-09-19

**Authors:** Yirga Legesse Niriayo, Gebre Teklemariam Demoz, Solomon Weldegebreal Asgedom, Gebremicheal Gebreyohannes Kahsay, Kidu Gidey

**Affiliations:** ^1^ Department of Clinical Pharmacy, School of Pharmacy, College of Health Sciences, Mekelle University, Mekelle, Tigray, Ethiopia, mu.edu.et; ^2^ Clinical Pharmacy and Pharmacy Practice Unit, Department of Pharmacy, College of Health Sciences, Aksum University, Aksum, Tigray, Ethiopia, aku.edu.et

**Keywords:** blood pressure control, Ethiopia, hypertension, self-care activities

## Abstract

**Background:** Adherence to self‐care activities is crucial for effective control of hypertension. However, little is known about the effect of self‐care activities on blood pressure control in Ethiopia. Hence, the present study is aimed at investigating the effect of self‐care activities on blood pressure control among patients with hypertension.

**Methods:** A prospective observational study was conducted among 326 hypertensive patients at a tertiary care hospital in Ethiopia. We adopted Hypertension Self‐Care Activity Level Effects (H‐SCALE) to assess adherence to self‐care activities. Data were collected through patient interviews and reviews of medical records. Binary logistic regression analysis was done to assess the effect of self‐care activities on blood pressure control.

**Result:** A total of 326 patients were included in the study. Blood pressure was controlled in 186 (57.1%) patients. In this study, 51.2%, 41.1%, 22.4%, and 25.2% of the patients were adherent to antihypertensive medications, the recommended physical activity level, weight management, and a low‐salt diet, respectively. The majority of the patients were nonsmokers (90.8%) and alcohol abstainers (72.1%). In multivariable binary logistic regression analysis, nonadherence to self‐care activities including medication intake (adjusted odds ratio [AOR]: 2.69, 95% confidence interval [CI]:1.61–4.51), weight management (AOR: 3.74, 95% CI: 1.92–7.32), low‐salt diet (AOR: 2.85, 1.55–5.24), and nonsmoking (AOR: 3.28, 95% CI: 1.12–9.64) were predictors of uncontrolled blood pressure.

**Conclusion:** Our findings indicated that a significant proportion of the participants had uncontrolled blood pressure. Participants with a low rate of adherence to self‐care activities, particularly weight management, low salt intake, nonsmoking, and medication intake, were more likely to have uncontrolled blood pressure. Therefore, we recommend further interventional programs aimed at enhancing these self‐care activities to achieve optimal BP control.

## 1. Introduction

Hypertension (HTN) is a major public health problem affecting over 1.3 billion people worldwide, and its burden is increasing most rapidly in developing regions, including Sub‐Saharan Africa [1–4]. A meta‐analysis conducted in Ethiopia in 2014 reported an overall HTN prevalence of 19.6% [5]. HTN remains one of the most important preventable contributors to cardiovascular morbidity and mortality [6–8]. In Ethiopia, HTN ranks among the top six causes of death, accounting for 3% of all‐cause mortality in 2005/2006 [9, 10].

Optimal blood pressure (BP) control is crucial to reduce cardiovascular complications and associated mortalities [11, 12]. Despite the use of effective antihypertensive medications, optimal BP control is not achieved, particularly in low‐ and middle‐income countries [13, 14]. Likewise, limited studies in Ethiopia have reported that BP was poorly controlled in the majority of hypertensive patients [15, 16]. Poorly controlled BP is a major risk factor for unfavorable outcomes such as stroke, heart failure, renal failure, and premature death [8, 17].

Effective hypertension management requires more than pharmacological interventions. The biopsychosocial model, proposed by Engel, emphasizes the integration of biological, psychological, and social factors in managing chronic conditions such as HTN [18]. In this context, self‐care activities including medication adherence, low‐salt diet, physical activity, weight management, alcohol abstinence, and smoking cessation are crucial for the prevention and control of HTN [11, 19]. However, motivating patients to achieve high self‐care activities adherence is challenging [20].

The health belief model (HBM) offers a useful framework for understanding and influencing adherence to self‐care activities [21]. According to HBM, a person’s engagement in health‐related actions is influenced by their perceptions of susceptibility, severity, benefits, and barriers, as well as cues to action and self‐efficacy. These components play a critical role in shaping how patients manage their hypertension through lifestyle modification and behavior change [22].

Despite the benefits of evidence‐based hypertension self‐care activities in BP control, adherence among hypertensive patients is generally low [15]. Nonadherence to self‐care activities is a leading contributor to poor BP control [23]. However, awareness and adherence remain low in African populations, including Ethiopia [24]. This gap highlights the need for context‐specific studies that explore the role of self‐care in HTN control. Therefore, the present study is aimed at investigating the impact of self‐care activities on BP control among hypertensive patients in Ethiopia.

## 2. Methods

### 2.1. Study Design and Study Setting

A prospective observational study was conducted from June 1, 2021, to February 28, 2022, at the cardiac clinic of Ayder Comprehensive Specialized Hospital (ACSH), the largest public hospital in Tigray, serving a catchment population of approximately 10 million people.

### 2.2. Study Population and Data Collection Procedure

All hypertensive patients aged ≥ 18 years who had been on follow‐up for at least 6 months with at least one antihypertensive medication were included in the study. Patients were excluded if they were too seriously ill to complete the interview, refused to provide consent, or had incomplete medical records.

A total of 350 patients were approached, of whom 326 were included in the study, while 24 were excluded due to unwillingness to participate [14], incomplete medical records [4], or inability to be interviewed due to serious illness [6]. Informed consent was obtained from all participants after explaining the purpose of the study. Patients were recruited during their medication refill appointments using a simple random sampling technique. Adherence to self‐care activities was initially assessed at baseline during the first 3 months of the study (June 1, 2021, to August 30, 2021). Enrolled patients were then required to return for follow‐up visits according to their schedule to assess BP control status. After the baseline assessment, all patients were followed for 6 months to evaluate their BP control status and its association with self‐care activities. BP was measured at every visit according to the scheduled follow‐up period. To determine BP control status, at least three consecutive follow‐up appointments, including baseline BP, were required for each patient. Patients whose BP remained controlled for at least three consecutive follow‐up visits were classified as having controlled BP.

The questionnaire was translated into the local language (Tigrigna) and then back‐translated into English to ensure consistency. The Tigrigna version of the questionnaire was evaluated for content validity by a panel of three experts in the field, including two from the Clinical Pharmacy Department and one from the Internal Medicine Department. The internal consistency reliability (Cronbach’s *α*) of the Tigrigna version was 0.81, indicating good reliability. A pretest was conducted on 5% of the sample (17 patients) at a different hospital, and the drafted questionnaire was assessed for face validity. Based on the findings, minor amendments were made to eliminate ambiguities and improve clarity.

Information on sociodemographics and adherence to self‐care activities was collected through patient interviews using a standardized questionnaire. Relevant medical and medication records, including working diagnoses, BP readings, and prescribed medications, were obtained from patients’ medical records using a data abstraction checklist. Fifth‐year clinical pharmacy students were employed as data collectors for this study. They received training on the study objectives, data collection methods, patient chart data extraction, and effective patient interviewing techniques.

### 2.3. BP Measurement

BP was measured at each scheduled follow‐up visit using a calibrated digital sphygmomanometer following international guidelines [25, 26]. Participants were seated comfortably with their back supported and feet flat on the floor, resting for at least 5 min before measurement. BP was measured on the same arm used in previous follow‐up visits, supported at heart level. For each visit, two readings were taken 1–2 min apart, and the average was recorded. If the two readings differed by more than 5 mmHg, a third measurement was obtained, and the average of the two closest values was used. Whenever possible, measurements were performed at approximately the same time of day to reduce diurnal variation.

To determine BP control status, at least three consecutive follow‐up appointments, including baseline BP, were required for each patient. Patients whose BP remained within the target range for at least three consecutive follow‐up visits were classified as having controlled BP.

### 2.4. Assessment and Scoring of Self‐Care Activities

We adopted Hypertension Self‐Care Activity Level Effects (H‐SCALEs) to assess patients’ adherence to self‐care activities. H‐SCALE has been validated for use in other similar studies [27–29]. It is a self‐reported questionnaire that consists of six categories of self‐care activities including medication adherence, low salt intake, physical activities, nonsmoking, weight management, and alcohol abstinence. Medication adherence includes three items scored by the number of days (0–7), with full adherence defined as taking medications exactly as prescribed every day of the week (a total score of 21). The low‐salt diet domain consists of six items also scored by days per week, with adherence defined as following the diet for at least 6 days. Physical activity is measured by two items, with adherence based on achieving either 150 min of moderate‐intensity exercise weekly or meeting the activity recommendation on six or more days. Nonsmoking is assessed by a single item, where adherence means no tobacco use in the past 7 days. Weight management includes 10 items rated on a 5‐point scale, with adherence defined as an average score of three or higher. Finally, alcohol abstinence is assessed by three items, with adherence defined as no alcohol consumption in the past week. Scoring and adherence cut‐offs were applied according to the H‐SCALE manual and supported by prior validation studies [28].

### 2.5. Data Analysis

Data were entered into Epi info (Version 7) and analyzed using the Statistical Package for the Social Sciences (SPSS Version 21.0). We checked multicollinearity among predictor variables using the variance inflation factor (VIF) and none were collinear. Univariable logistic regression analysis was done to determine the association of each independent variable with BP control. Furthermore, independent variables with *p* < 0.25 in the univariable binary logistic regression analysis were re‐entered into a multivariable binary logistic regression model to identify predictors of BP control. A *p* value of < 0.05 was considered statistically significant in all analyses.

### 2.6. Operational Definitions

Adherence to self‐care cavities is the extent of the patient’s adherence to the six categories of self‐care activities, including medication adherence, physical activity, alcohol abstinence, nonsmoking, low salt intake, and weight management, which are measured by H‐SCALE [28]. Categories of self‐care activities were defined and measured based on H‐SCALE [28] as follows: Adherent to medication: a patient who took the prescribed antihypertensive medications at the same time every day and took the recommended dose for 7 out of 7 days; adherent to low‐salt diet: a patient who took salt‐free diet while cooking and eating for at least 6 or better days out of 7 days; adherent to physical activity: a person who did physical activity for at least 30 min per day for 8 or better days out of 14 days; alcohol abstainer: a patient who did not drink any alcohol in the last 7 days or who reported that he/she usually did not drink at all; nonsmoker: a patient who did not smoke any pack of cigarettes in the last 7 days or who indicated that he/she usually did not smoke at all; and adherent to weight management: this is assessed with 10 items related to dietary practices such as reducing portion size and making food substitutions, as well as exercising to lose weight. The items assessed agreement with weight management activities during the past 30 days. Response categories use a 5‐point Likert scale ranging from strongly disagree [1] to strongly agree [5], with scores ranging from 10 to 50. Participants who scored 40 and above out of 50 were said to be adherent to weight management.

Controlled BP was defined as an average systolic BP < 140 and diastolic BP < 90 mmHg for at least three consecutive follow‐up appointments if the patient is younger than 60 years old or an average systolic BP < 150 and diastolic BP < 90 mmHg if the patient was 60 years old and above; otherwise, it was said to be uncontrolled BP (31).

Khat (*Catha edulis*) is a green leafy plant cultivated throughout Eastern Africa and Arab countries. It is a natural stimulant that contains psychoactive substances cathinone and cathine, which have effects like amphetamines [30, 31].

## 3. Results

### 3.1. Sociodemographic Characteristics

A total of 326 participants were included in the study. The mean (± standard deviation (SD) age of the patients was 53.00 ± 13.66 years, and 55.5% were females. More than half (55.2%) of the participants were married, 78.2% were from urban areas, and 37.4% were unable to write and read (Table 1).

**Table 1:** Sociodemographic characteristics of patients with hypertension in Tigray.

**Characteristics**	**Total,** **n** **(%)**	**Blood pressure**		**p** **value**
		**Controlled,** **n** **(%)**	**Uncontrolled,** **n** **(%)**	
Gender				
Female	181 (55.5)	109 (60.2)	72 (39.8)	0.197
Male	145 (44.5)	77 (53.1)	68 (46.9)	
Age in years				
18–35	32 (9.8)	15 (46.9)	17 (53.1)	0.224
36–60	184 (56.4)	102 (55.4)	82 (44.6)	
> 60	110 (33.7)	69 (62.7)	41 (37.3)	
Residence				
Urban	255 (78.2)	141 (55.3)	114 (44.7)	0.223
Rural	71 (21.8)	45 (63.4)	26 (36.6)	
Educational level				
Unable to write and read	122 (37.4)	74 (60.7)	48 (39.3)	0.712
Primary education	86 (26.4)	48 (55.8)	38 (44.2)	
Secondary education	45 (13.8)	23 (51.1)	22 (48.9)	
College and above	73 (22.4)	41 (56.2)	32 (43.8)	
Marital status				
Married	180 (55.2)	100 (55.6)	80 (44.4)	0.590
Single	68 (20.9)	42 (61.8)	26 (38.2)	
Divorced	36 (11.0)	18 (50.0)	18 (50.0)	
Widowed	42 (12.9)	26 (61.9)	16 (38.1)	
Occupation				
Government employee	57 (17.5)	32 (56.1)	25 (43.9)	0.646
Nongovernment employee	39 (12.0)	20 (51.3)	19 (48.7)	
Merchant	92 (28.2)	53 (57.6)	39 (42.4)	
Farmer	40 (12.3)	27 (67.5)	13 (32.5)	
Housewife	98 (30.1)	54 (55.1)	44 (44.9)	
Khat chewing				
No	28 (8.6)	175 (58.7)	123 (41.3)	0.051
Yes	298 (91.4)	11 (39.3)	17 (60.7)	

### 3.2. Clinical and Treatment‐Related Factors

More than half (55.8%) of the patients had one or more comorbidities and 42.3% of patients had lived with HTN for ≥ 5 years. The mean number of medications per patient was 2.63 ± 1.28 and 41.7% were taking at least three medications (Table 2).

**Table 2:** Clinical and treatment related characteristics of patients with hypertension in Tigray.

**Characteristics**	**Total,** **n** **(%)**	**Blood pressure**		**p** **value**
		**Controlled,** **n** **(%)**	**Uncontrolled,** **n** **(%)**	
Comorbidity				
No	144 (44.2)	77 (55)	63 (45.0)	0.515
Yes	182 (55.8)	109 (58.6)	77 (41.4)	
Duration of hypertension				
< 5year	188 (57.7)	106 (56.4)	82 (43.6)	0.775
≥ 5year	138 (42.3)	80 (58.0)	58 (42.0)	
Number of medications				
< 3	190 (58.3)	105 (55.3)	85 (44.7)	0.440
≥ 3	136 (41.7)	81 (59.6)	55 (40.4)	

Abbreviation: HCT, hydrochlorothiazide.

The most commonly used antihypertensive monotherapy was hydrochlorothiazide (HCT) (13.5%), followed by nifedipine (9.5%) and enalapril (8.3%) while HCT + enalapril (25.5%) and HCT + nifedipine (24.2%) were the most commonly used combination therapies (Table 3).

**Table 3:** Utilization of antihypertensive medications among patients with hypertension in Tigray.

**Utilization of antihypertensive medications**	**n** **(%)**
HCT	44 (13.5)
Nifedipine	31 (9.5)
Enalapril	27 (8.3)
Amlodipine	20 (6.1)
HCT + enalapril	83 (25.5)
HCT + nifedipine	79 (24.2)
Enalapril + nifedipine	26 (8.0)
HCT + amlodipine	13 (40)
Atenolol +enalapril	7 (2.5)
HCT + enalapril + nifedipine	6 (2.2)
HCT + enalapril +atenolol	5 (1.8)

### 3.3. Adherence to Self‐Care Activities

Our result indicated that 51.2% of the patients were adherent to their antihypertensive medications. Only 41.1%, 22.4%, and 25.2% of patients were adherent to the recommended physical activity level, weight management, and low‐salt diet, respectively. The majority were nonalcohol consumers (72.1%) and nonsmokers (90.8%) (Table 4).

**Table 4:** Self‐care activities among patients with hypertension in Tigray.

**Self-care activities**	**Total,** **n** **(%)**	**Blood pressure**		**p** **value**
		**Controlled,** **n** **(%)**	**Uncontrolled,** **n** **(%)**	
Medication adherence^a^				
Yes	167 (51.2)	107 (64.1)	60 (35.9)	0.009
No	159 (48.8)	79 (49.7)	80 (50.3)	
Low‐salt diet adherence^b^				
Yes	82 (25.2)	61 (74.4)	21 (25.6)	0.001
No	244(74.8)	126 (51.6)	118 (48.4)	
Weight management^c^				
Yes	73 (22.4)	56 (76.7)	17 (23.3)	< 0.001
No	253(77.6)	130 (51.4)	123 (48.6)	
Physical activity^d^				
Yes	134 (41.1)	84 (62.7)	50 (37.3)	0.086
No	192 (58.9)	102 (53.1)	90 (46.9)	
Alcohol use^e^				
Abstainer	235 (72.1)	139 (59.1)	96 (40.9)	0.220
Drinker	91 (27.9)	47 (51.6)	44 (48.4)	
Smoking status^f^				
Nonsmoker	296(90.8)	178 (60.1)	118 (39.9)	< 0.001
Smoker	30 (9.2)	8 (26.7)	22 (73.3)	

^a^Patient who took the prescribed medications at the same time every day and took the recommended dose for 7 out of 7 days is said to be adherent to medication.

^b^Patient who took salt‐free diet while cooking and eating for at least 6 or better days out of 7 days is said to be adherent to low‐salt diet.

^c^Weight management refers to a set of practices and behaviors that are necessary to keep one’s weight at a healthful level, including reducing the amount of food portion size, making food substitutions, and exercising to lose weight.

^d^Patient who did physical activity for at least 30 min per day for 8 or better days out of 14 days is said to be adherent to physical activity.

^e^Patient who did not drink any alcohol in the last 7 days or who reported that he/she usually did not drink at all is said to be an alcohol abstainer.

^f^Patient who did not smoke any pack of cigarettes in the last 7 days or who indicated that he/she usually did not smoke at all is said to be a nonsmoker.

### 3.4. BP Control Status

The mean (± SD) systolic BP reading was 134.11 ± 21.76 mmHg, ranging from 90.00 to 200.00 mmHg while the mean (± SD) diastolic BP reading was 86.44 ± 12.21 mmHg, ranging from 60.00 to 120.00 mmHg. Overall, 186 (57.1%) of the patients had optimally controlled BP.

### 3.5. Factors Related to BP Control

Univariable logistic regression analysis was performed to compare hypertensive patients with controlled and uncontrolled BP using the sociodemographics, self‐care activities, disease, and medication‐related characteristics. Accordingly, nonadherence to the prescribed medication (crude odds ratio (COR): 1.80, 95% confidence interval [CI]:1.16‐2.81), lack of weight management (COR:3.12, 95% CI:1.72–5.66), nonadherence to a low‐salt diet (COR:2.55, 95% CI: 1.48–4.42), and smoking (COR:4.15, 95% CI: 1.79–9.63) were significantly associated with uncontrolled BP (Table 5).

**Table 5:** Univariable logistic regression analysis of factors associated with BP control among patients with hypertension in Tigray.

**Predictors**	**Blood pressure**		**COR (95% CI)**
	**Controlled,** **n** **(%)**	**Uncontrolled,** **n** **(%)**	
Gender			
Female	109 (60.2)	72 (39.8)	1
Male	77 (53.1)	68 (46.9)	1.34 (0.86–2.08)
Age in year			
18–35	15 (46.9)	17 (53.1)	1
36–60	102 (55.4)	82 (44.6)	0.71 (0.33–1.51)
> 60	69 (62.7)	41 (37.3)	0.52 (0.24–1.16)
Residence			
Urban	141 (55.3)	114 (44.7)	1
Rural	45 (63.4)	26 (36.6)	0.72 (0.42–1.24)
Educational level			
Unable to write and read	74 (60.7)	48 (39.3)	0.83 (0.46–1.50)
Primary education	48 (55.8)	38 (44.2)	1.01 (0.54–1.90)
Secondary education	23 (51.1)	22 (48.9)	1.23 (0.58–2.58)
College and above	41 (56.2)	32 (43.8)	1
Marital status			
Married	100 (55.6)	80 (44.4)	1
Single	42 (61.8)	26 (38.2)	0.77 (0.44–1.37)
Divorced	18 (50.0)	18 (50.0)	1.25 (0.61–2.56)
Widowed	26 (61.9)	16 (38.1)	0.77 (0.39–1.53)
Occupation			
Government employee	32 (56.1)	25 (43.9)	1
Nongovernment employee	20 (51.3)	19 (48.7)	1.22 (0.54–2.75)
Merchant	53 (57.6)	39 (42.4)	0 0.94 (0.48–1.84)
Farmer	27 (67.5)	13 (32.5)	0.62 (0.27–1.43)
Housewife	54 (55.1)	44 (44.9)	1.04 (0.54–2.01)
Comorbidity			
No	77 (55)	63 (45.00)	1
Yes	109 (58.6)	77 (41.4)	0.86 (0.56‐–1.34)
Number of medications			
< 3	105 (55.3)	85 (44.7)	1
≥ 3	81 (59.6)	55 (40.4)	0.84 (0.54–1.31)
Khat chewing			
No	175 (58.7)	123 (41.3)	1
Yes	11 (39.3)	17 (60.7)	2.20 (0.10‐4.86)
Duration of diagnosis			
<5 years	106 (56.4)	82 (43.6)	1
≥ 5 years	80 (58)	58 (42)	0.94 (0.60–1.46)
Alcohol^e^			
Abstainer	139 (59.1)	96 (340.9)	1
Drinker	47 (51.6)	44 (48.4)	1.36 (0.83–2.21)
Smoking status^f^			
Nonsmoker	178 (69.3)	118 (30.7)	1
Smoker	8 (26.7)	22 (73.3)	4.15 (1.79–9.63)
Physical activity^d^			
Yes	84 (62.7)	50 (37.3)	1
No	102 (53.1)	90 (46.9)	1.48 (0.95–2.33)
Weight management^c^			
Yes	56 (76.7)	17 (23.3)	1
No	130 (51.4)	123 (48.6)	3.12 (1.72–5.66)
Low‐salt diet adherence^b^			
Yes	61 (74.4)	21 (25.6)	1
No	126 (51.6)	118 (48.4)	2.55 (1.48–4.42)
Medication adherence^a^			
Yes	107 (64.1)	60 (35.9)	1
No	79 (49.7)	80 (50.3)	1.80 (1.16–2.81)

Abbreviations: CI, confidence interval; COR, crude odds ratio.

^a^Patient who took the prescribed medications at the same time every day and took the recommended dose for 7 out of 7 days is said to be adherent to medication.

^b^Patient who took salt free diet while cooking and eating for at least 6 or better days out of 7 days is said to be adherent to low‐salt diet.

^c^Weight management refers to a set of practices and behaviors that are necessary to keep one’s weight at a healthful level including reducing the amount of food portion size, making food substitutions as well as exercising to lose weight.

^d^Patient who did physical activity at least 30 min per day for 8 or better days out of 14 days is said to be adherent to physical activity.

^e^Patient who did not drink any alcohol in the last 7 days or who reported that he/she usually did not drink at all is said to be alcohol abstainer.

^f^Patient who did not smoke any pack of cigarette in the last 7 days or who indicated that he/she usually did not smoke at all is said to be nonsmoker.

Furthermore, variables with *p* < 0.25 in the univariable analyses were included in the multivariable logistic regression model. The full model containing all variables was statistically significant (Chi‐square = 44.998, df = 10, *p* < 0.001). Independent predictors of uncontrolled BP were nonadherence to medication (AOR: 2.69, 95% CI: 1.61–4.51), lack of weight management (AOR: 3.74, 95% CI: 1.92–7.32), nonadherence to a low‐salt diet (AOR: 2.85, 95% CI: 1.55–5.24), and smoking (AOR: 3.28, 95% CI: 1.12–9.64) (Table 6).

**Table 6:** Multivariable logistic regression analysis of factors associated with BP control among patients with hypertension in Tigray.

**Predictors**	**Blood pressure control status**		**AOR (95% CI)**
	**Controlled,** **n** **(%)**	**Uncontrolled,** **n** **(%)**	
Gender			
Female	109 (60.2)	72 (39.8)	1
Male	77 (53.1)	68 (46.9)	1.38 (0.83–2.31)
Age in year			
18–35	15 (46.9)	17 (53.1)	1
36–60	102 (55.4)	82 (44.6)	0.52 (0.22–1.23)
> 60	69 (62.7)	41 (37.3)	0.42 (0.16–1.05)
Residence			
Urban	141 (55.3)	114 (44.7)	1
Rural	45 (63.4)	26 (36.6)	0.65 (0.34–1.23)
Khat chewing			
No	175 (58.7)	123 (41.3)	1
Yes	11 (39.3)	17 (60.7)	1.01 (0.34–2.99)
Alcohol^e^			
Abstainer	139 (59.1)	96 (34.9)	1
Drinker	47 (51.6)	44 (48.4)	1.67 (0.94–2.96)
Smoking status^f^			
Non‐smoker	178 (69.3)	118 (30.7)	1
Smoker	8 (26.7)	22 (73.3)	3.28 (1.12–9.64)
Physical activity^d^			
Yes	84 (62.7)	50 (37.3)	1
No	02 (53.1)	90 (46.9)	1.55 (0.91–2.62)
Weight management^c^			
Yes	56 (76.7)	17 (23.3)	1
No	130 (51.4)	123 (48.6)	3.74 (1.92–7.32)
Low‐salt diet adherence^b^			
Yes	61 (74.4)	21 (25.6)	1
No	126 (51.6)	118 (48.4)	2.85 (1.55–5.24)
Medication adherence^a^			
Yes	107 (64.1)	60 (35.9)	1
No	79 (49.7)	80 (50.3)	2.69 (1.61–4.51)

Abbreviations: AOR, adjusted odds ratio; CI, confidence interval.

^a^Patient who took the prescribed medications at the same time every day and took the recommended dose for 7 out of 7 days is said to be adherent to medication.

^b^Patient who took salt free diet while cooking and eating for at least 6 or better days out of 7 days is said to be adherent to low‐salt diet.

^c^Weight management refers to a set of practices and behaviors that are necessary to keep one’s weight at a healthful level including reducing the amount of food portion size, making food substitutions as well as exercising to lose weight.

^d^Patient who did physical activity at least 30 min per day for 8 or better days out of 14 days is said to be adherent to physical activity.

^e^Patient who did not drink any alcohol in the last 7 days or who reported that he/she usually did not drink at all is said to be alcohol abstainer.

^f^Patient who did not smoke any pack of cigarette in the last 7 days or who indicated that he/she usually did not smoke at all is said to be nonsmoker.

## 4. Discussion

### 4.1. BP Control and Its Association With Self‐Care Activities

Assessment of BP control and its association with self‐care activities is crucial for reducing the global burden of hypertension, particularly in developing countries [32]. Therefore, our study determined the rate of BP control and its association with self‐care activities. Our study revealed that 42.9% of the patients had uncontrolled BP. The majority of participants were neither alcohol consumers nor smokers. However, less than half of the participants were adherent to the recommended level of physical activity, low salt intake, and weight management. However, about half (51.2%) of the participants were adherent to the prescribed medications.

Although optimal BP control significantly reduces morbidity and mortality associated with hypertension, it remains poorly controlled worldwide [3, 13]. Several studies reported that the rate of BP control was low across African regions including Ethiopia owing to the poor healthcare facilities and low level of awareness about HTN management [33, 34]. Likewise, a significant proportion of the patients had uncontrolled BP in our study. In the present study, BP was controlled in 57.1% of the patients, which is in line with a finding from the Ghanaian study, 57.7% [35]. In contrast, the rate of BP control in the present study was higher compared to other similar studies conducted in Uganda, 9.4%, Kenya, 33.4%, Cameroon, 36.8%, and Debretabor, Ethiopia, 42.9% [36–39]. This could be attributed to the difference in the level of healthcare facilities and medical practitioners’ expertise. For instance, our study was conducted in a tertiary care hospital while the aforementioned studies [33, 34, 37, 39] were conducted at lower levels of healthcare facilities including primary care, district, and general hospitals. Therefore, patients treated at the tertiary care level might have a higher rate of controlled BP than those treated in the lower levels of healthcare facilities as tertiary care hospitals might have more qualified healthcare professionals and advanced equipment. Moreover, patient‐related factors including sociodemographics, cultural beliefs, educational level, and awareness about the disease and treatment might have contributed to the differences. In our study, higher proportions (78%) of the patients were from urban areas compared to the aforementioned studies [33, 34, 37, 39]. Hence, the employment of a higher proportion of rural residents in these studies might have led to the low rate of adherence to the prescribed medications and other self‐care activities owing to the poor health literacy, lack of access to healthcare, and mistrust of modern medicine in the rural population [40, 41] which in turn led to a higher rate of uncontrolled BP.

According to our survey, nonadherence to self‐care activities such as low salt intake, weight management, medication intake, and smoking cessation was positively associated with uncontrolled BP. Among the significantly associated self‐care activities, smoking (AOR: 3.28, 95% CI: 1.12–9.64) and poor weight management (AOR: 3.74, 95% CI: 1.92–7.32) were more strongly associated with BP control than medication non‐adherence (AOR: 2.69, 95% CI: 1.61–4.51) and nonadherence to low salt intake (AOR: 2.85, 1.55–5.24). In line with our study, other similar studies conducted in developed and developing countries including Ethiopia reported that poor weight management, excess salt intake, medication nonadherence, and smoking were positively associated with uncontrolled BP [15, 36, 42–44]. These findings can be explained by the HBM, which suggests that patients’ engagement in health behaviors depends on their perceptions of risk, benefits, and barriers [21]. Those with better BP control likely recognize their susceptibility to hypertension complications and the benefits of self‐care, while overcoming obstacles such as cost or cultural beliefs. Conversely, poor adherence may reflect low perceived risk or significant barriers [18, 45]. Interventions guided by the HBM focusing on increasing awareness of risks, emphasizing benefits, and reducing barriers could enhance adherence and improve BP control in this population [46].

### 4.2. Medication Adherence and BP Control

Among the self‐care activities, medication adherence is the most important activity for effective control of HTN. However, nearly half (48.8%) of participants were not adherent to their medications in the present study, which is consistent with a finding reported from Nigeria, 49% [47]. In contrast, a lower rate of nonadherence was reported from Italy, 25% [48]. The possible reason for the higher rate of nonadherence to medication in our study could be due to inadequate health literacy, inability to afford medicine, high trust in traditional medicine, lack of access to healthcare, and poor perception of patients toward illnesses and medication in developing countries including Ethiopia [34, 49–51]. Moreover, medication nonadherence was positively associated with uncontrolled BP in our study. Similarly, medication nonadherence was positively associated with uncontrolled BP in Ghanaian and Cameroon studies [36, 52]. Therefore, more effort needs to be done to improve medication adherence and BP control.

### 4.3. Low‐Salt Dietary Adherence and BP Control

Eating a low‐salt diet is crucial for controlling BP and reducing associated cardiovascular complications [24, 53]. However, only one‐fourth of participants were adherent to low‐salt diet recommendations in the present study, which was in line with an African–American study in which only 22% were adherent to low‐salt diet recommendations [27]. This could be attributed to unhealthy feeding habits, difficulty in restricting salt when cooking traditional food, and poor self‐care habits of the African population, including Ethiopia [54, 55]. Our study indicated that participants who were not adherent to a low‐salt diet were more likely to have uncontrolled BP compared to the adherent ones. This finding is consistent with previous studies conducted in Cameroon [36] and Gonder, Ethiopia [15] in which excess salt intake was inversely associated with BP control.

### 4.4. Weight Management and Physical Activity in BP Control

Despite the fact that effective weight management has a positive impact on BP control and cardiovascular risk reduction [56, 57], a low rate of adherence to weight management was reported in our study. Similar findings were also reported from African–American and Iranian studies [27, 58]. More importantly, we found a significant positive association between weight management and BP control in the current study. This finding is supported by a previous study in Ethiopia [59]. The potential barriers to following good weight management practices in Africa, including Ethiopia, could be poor awareness of hypertension management, poor access to weight management guidelines and facilities, and misconceptions about exercise and weight gain [55, 59]. Therefore, interventional programs, including education, might be helpful to promote hypertension self‐care activities, including effective weight management practices and attaining better BP control [60]. Although several studies demonstrated that physical inactivity resulted in increased BP and its complications [36, 61], physical inactivity was not significantly associated with the rate of BP control in this study. These variations could be attributed to the differences in study design, clinical characteristics, and population demographics.

### 4.5. Smoking and Alcohol Consumption in BP Control

Consistent with earlier Ethiopian studies [15, 62], the majority of participants in our study (90.8%) were nonsmokers. By contrast, a considerably higher smoking prevalence has been reported among African–American populations [27], which may reflect the lower sociocultural acceptance of cigarette smoking in Ethiopia [54]. In line with other scientific studies [63, 64], smoking was significantly associated with poor control of BP in our study. Therefore, encouraging patients to reduce or quit smoking could be helpful to improve BP control [63]. Although alcohol use has been implicated in raising BP and contributing to cardiovascular complications in various studies [65, 66], we did not find evidence of a significant effect on BP control in our sample. This may be due to lower consumption levels or patterns in the study population that limit its measurable effect on BP control. Alternatively, the impact of alcohol could be masked by stronger influences from other behavioral or clinical factors in this population.

### 4.6. Limitations

This study is not without limitations. Patients may understate socially undesirable activities like non‐adherence to self‐care activities. Information related to self‐care activities adherence was collected by interview, which might lead to recall bias. Although we have attempted to assess various factors that might influence BP control, we did not assess the impact of health professionals level of knowledge and clinical inertia on BP control. Moreover, as the findings of this study could be affected by the differences in participants’ characteristics, disease distribution, healthcare infrastructure, and methods employed, they should be extrapolated to other countries with caution. Future studies should consider incorporating more objective measures of adherence, such as pill counts, pharmacy refill records, or 24‐h urinary sodium excretion, to reduce reporting bias and enhance measurement accuracy.

## 5. Conclusion

In this study, a significant proportion of patients had uncontrolled BP. Patients who were not adherent to self‐care activities like weight management, low salt intake, nonsmoking, and medication intake were more likely to have uncontrolled BP. Therefore, we recommend further interventional programs with the aim of enhancing these self‐care activities to achieve optimal BP control. In alignment with Ethiopia’s National noncommunicable disease strategy and health promotion initiatives, these interventions should be integrated into community‐based programs and primary healthcare services, with an emphasis on patient education, behavior change support, and regular monitoring. Moreover, we recommend that researchers conduct further longitudinal studies with robust designs to better establish the cause‐and‐effect relationship between self‐care activities and BP control.

**Nomenclature**
AOR: adjusted odds ratio
BP: blood pressure
CI: confidence interval
CVD: cardiovascular disease
H‐SCALE: hypertension self‐care activity level effect
HTN: hypertension
SPSS: Statistical Package for Social Science

## Ethics Statement

Approval for this study was obtained from the Ethics Review Committee of the School of Pharmacy, College of Health Sciences, Mekelle University. The aim and protocol of the study were fully explained to each participant included in the study. Written informed consent was obtained from each participant. The personal information was entirely confidential and protected, and all methods were performed in accordance with the approved institutional guidelines.

## Consent

The authors have nothing to report.

## Disclosure

All authors have approved the final version of the manuscript for submission.

## Conflicts of Interest

The authors declare no conflicts of interest.

## Author Contributions

Y.L.N. conceptualized and designed the study, analyzed and interpreted the data, and drafted the original manuscript. G.T.D., S.W.A., G.G.K., and K.G. assisted in study design, data analysis, and manuscript evaluation. All authors have made intellectual contributions.

## Funding

No funding was received for this manuscript.

## Data Availability

All data related to this article will be accessible on reasonable request from the corresponding author.
